# Supplementary figures and images for: Immunoglobulin J chain as a non-invasive indicator of pregnancy in the cheetah (Acinonyx jubatus)
Source: PLoS One. 2020 Feb 10;15(2):e0225354. doi: 10.1371/journal.pone.0225354 (PMC7010269; doi:10.1371/journal.pone.0225354)

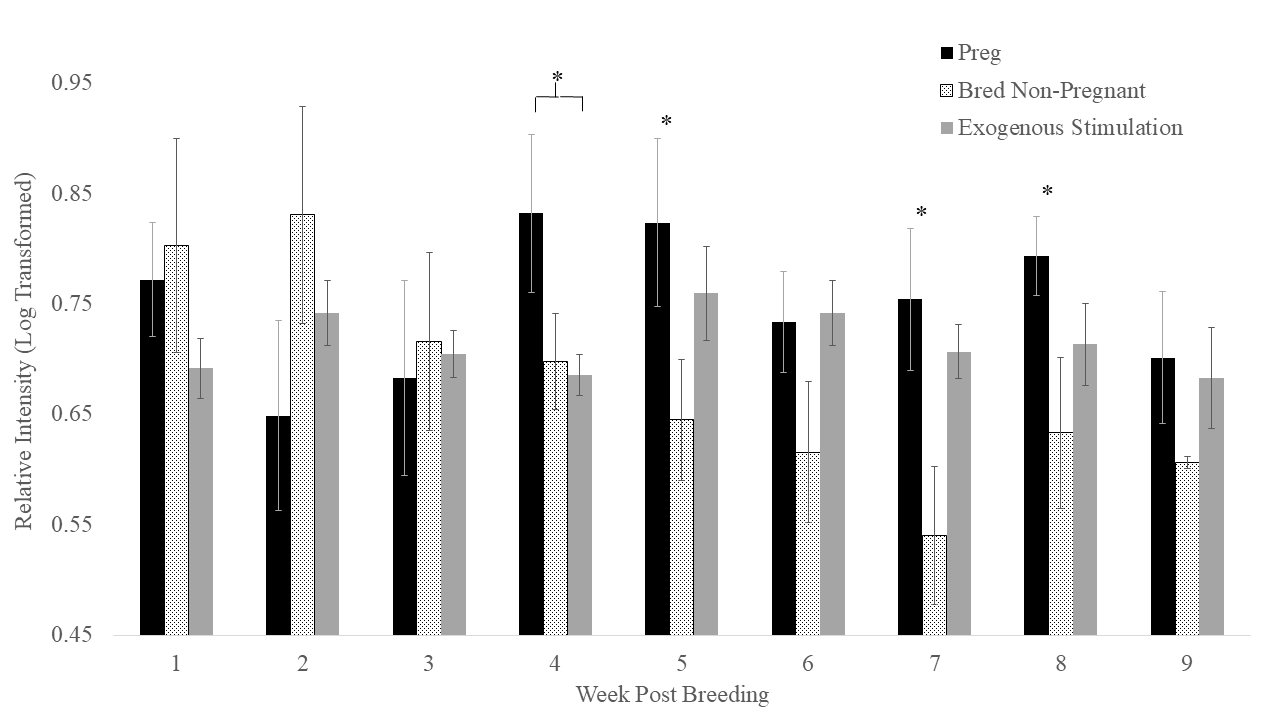

Supplement: S1 Fig — (TIF) [file pone.0225354.s001.tif]
