# Supplementary material for: Immunoglobulin J chain as a non-invasive indicator of pregnancy in the cheetah (Acinonyx jubatus)
Source: PLoS One. 2020 Feb 10;15(2):e0225354. doi: 10.1371/journal.pone.0225354 (PMC7010269; doi:10.1371/journal.pone.0225354)

SW.1

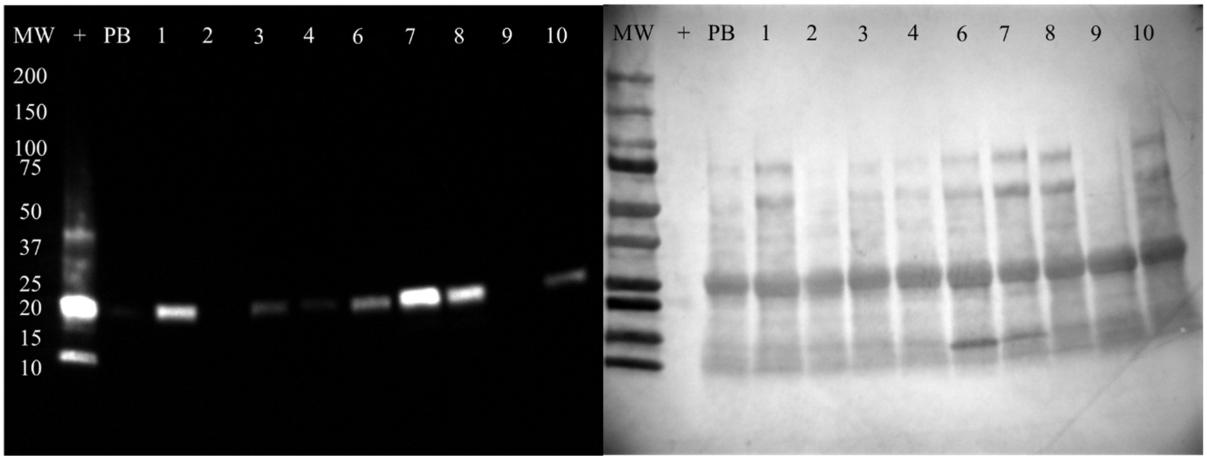

MTa.1

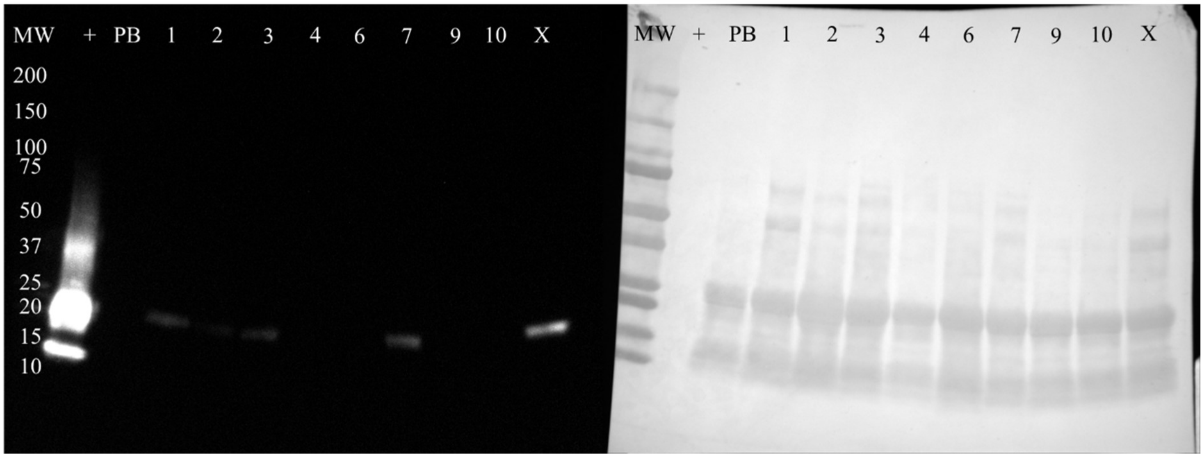

ZZ.2a

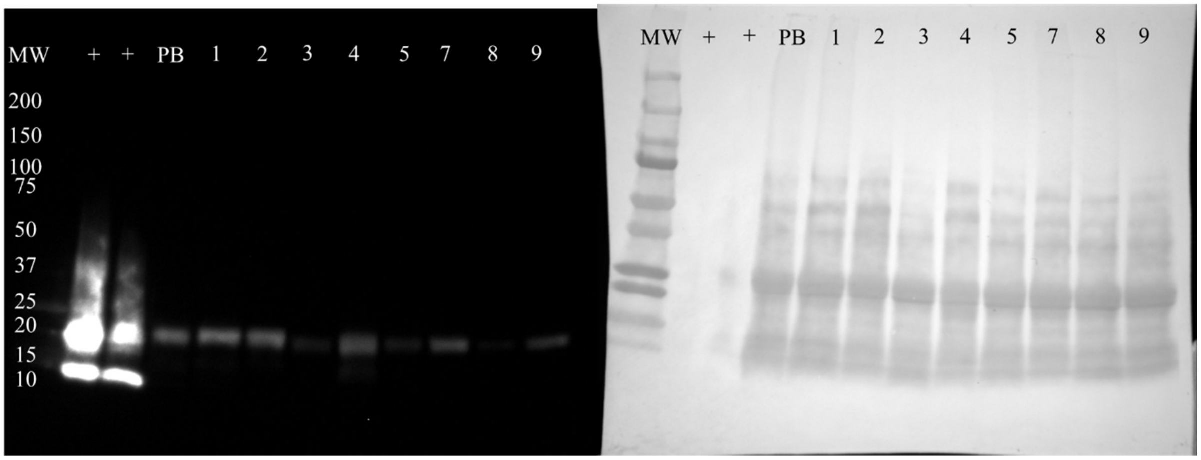

SN.1

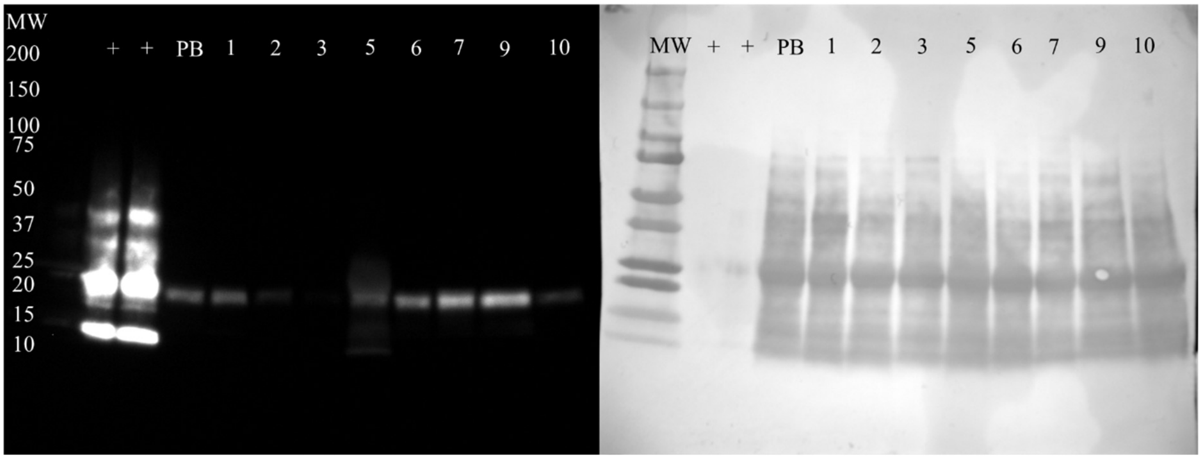
MW
200
150
100
75
50
37
25
20
15
10
+ + PB 1 2 3 5 6 7 9 10
MW + + PB 1 2 3 5 6 7 9 10

ZZ.1

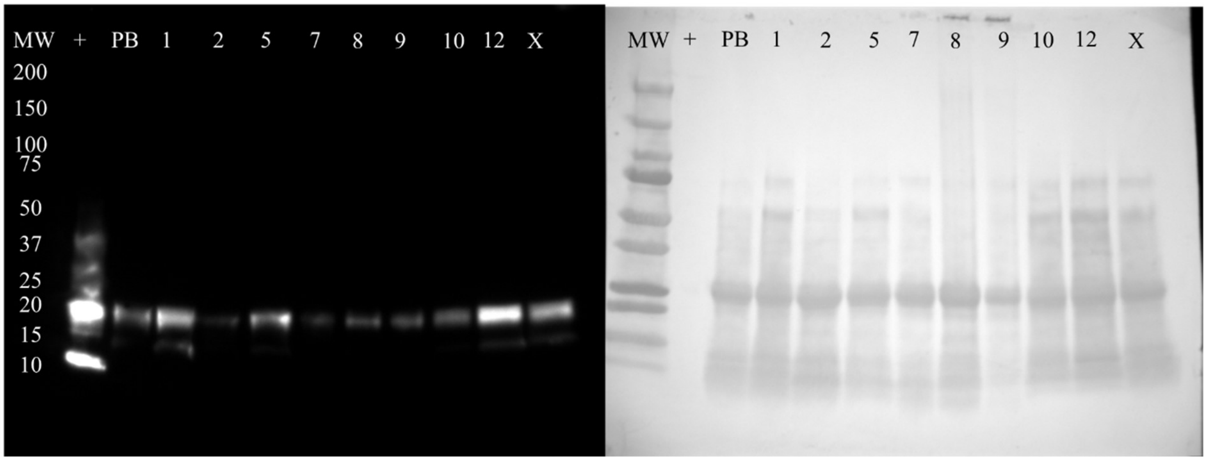
MW + PB 1 2 5 7 8 9 10 12 X
200
150
100
75
50
37
25
20
15
10
MW + PB 1 2 5 7 8 9 10 12 X

ALa.1

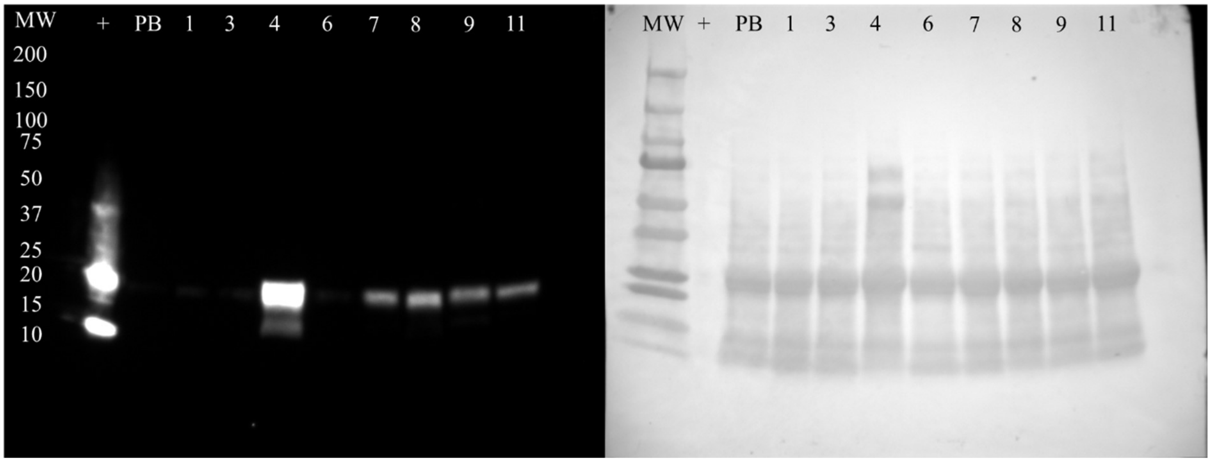
MW
+ PB 1 3 4 6 7 8 9 11
200
150
100
75
50
37
25
20
15
10
MW + PB 1 3 4 6 7 8 9 11

AL.1

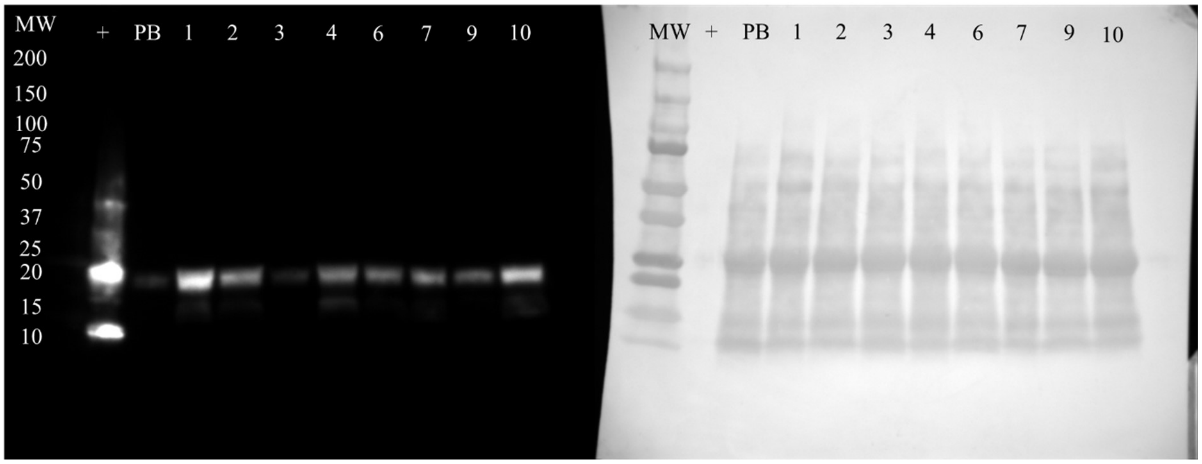
MW + PB 1 2 3 4 6 7 9 10
200
150
100
75
50
37
25
20
15
10
MW + PB 1 2 3 4 6 7 9 10

SV.3

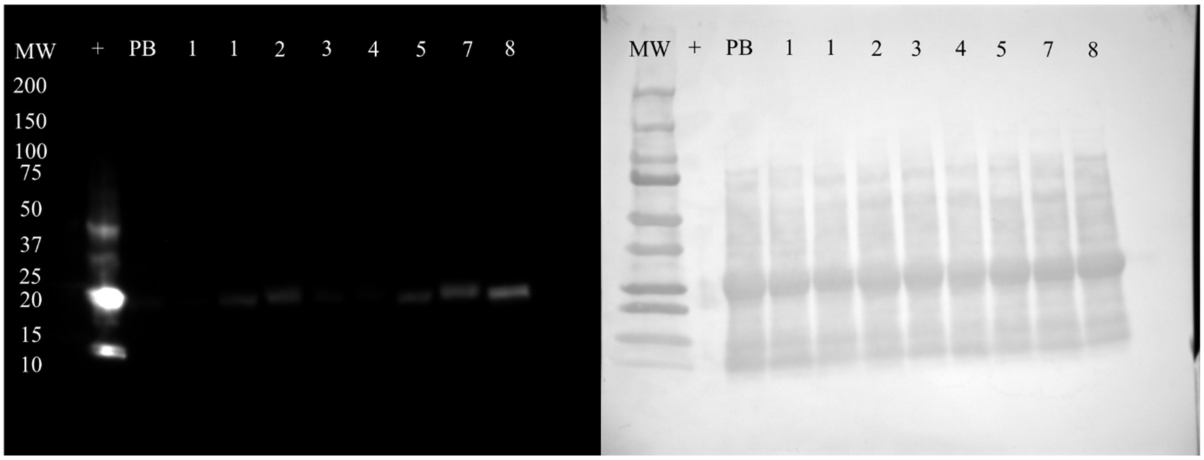
MW + PB 1 1 2 3 4 5 7 8
200
150
100
75
50
37
25
20
15
10
MW + PB 1 1 2 3 4 5 7 8

DM.3

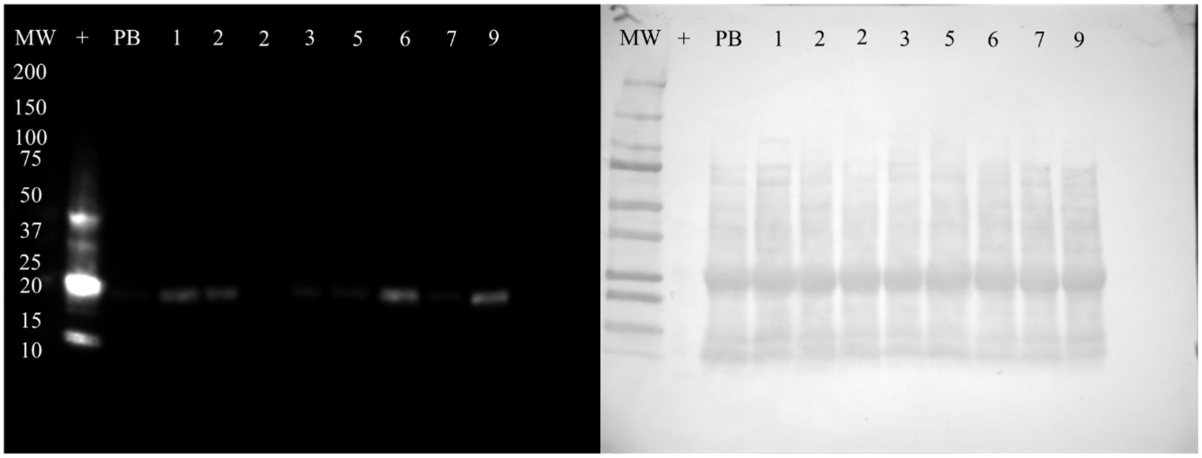
MW + PB 1 2 2 3 5 6 7 9
200
150
100
75
50
37
25
20
15
10
MW + PB 1 2 2 3 5 6 7 9

NKb.1

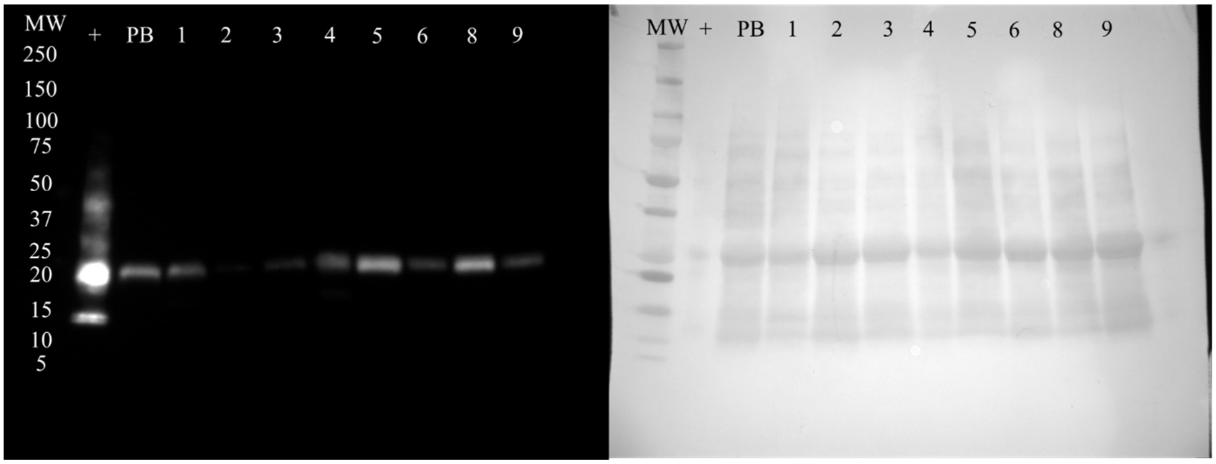

PA.1

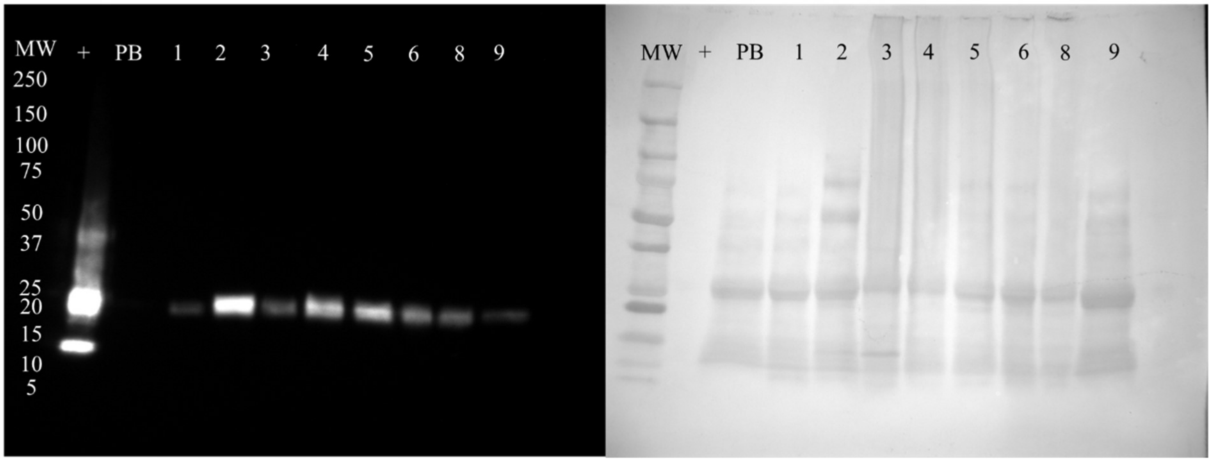

TM.2b

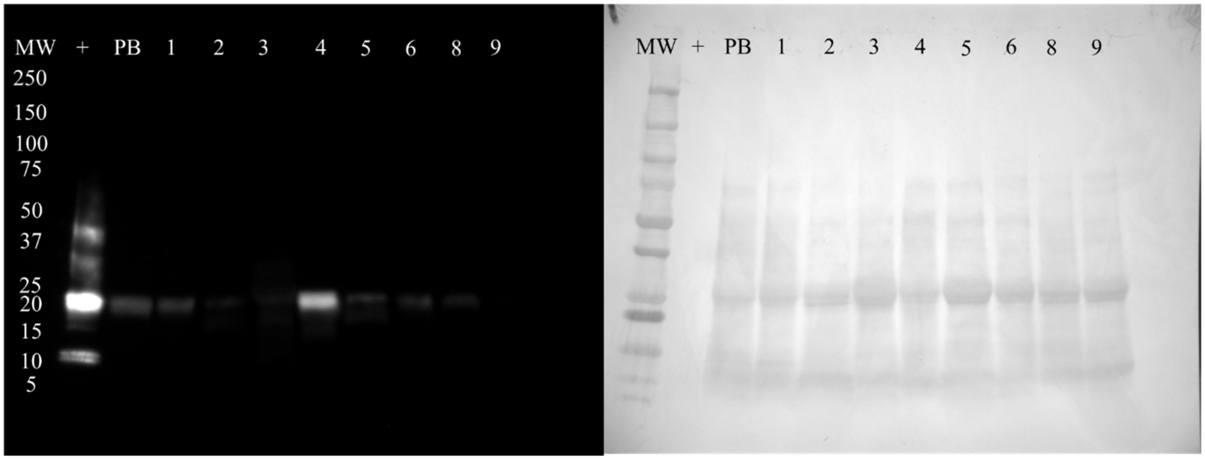

NOa.2a

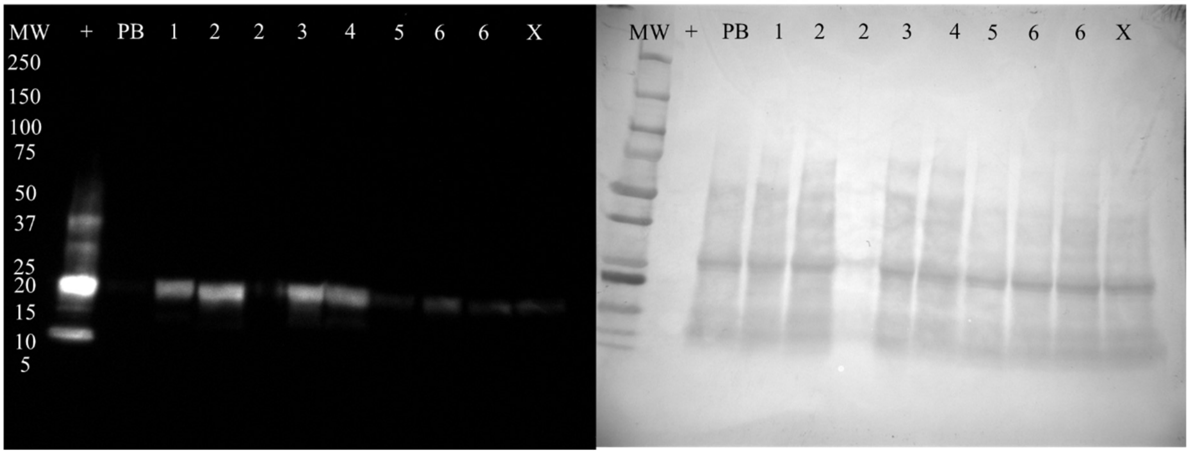

SW.2a

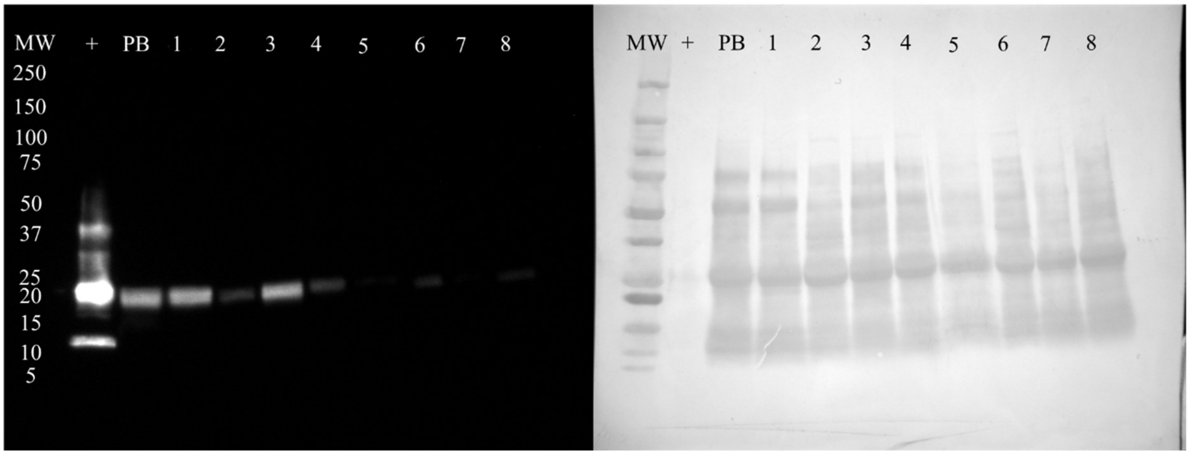

AM.1

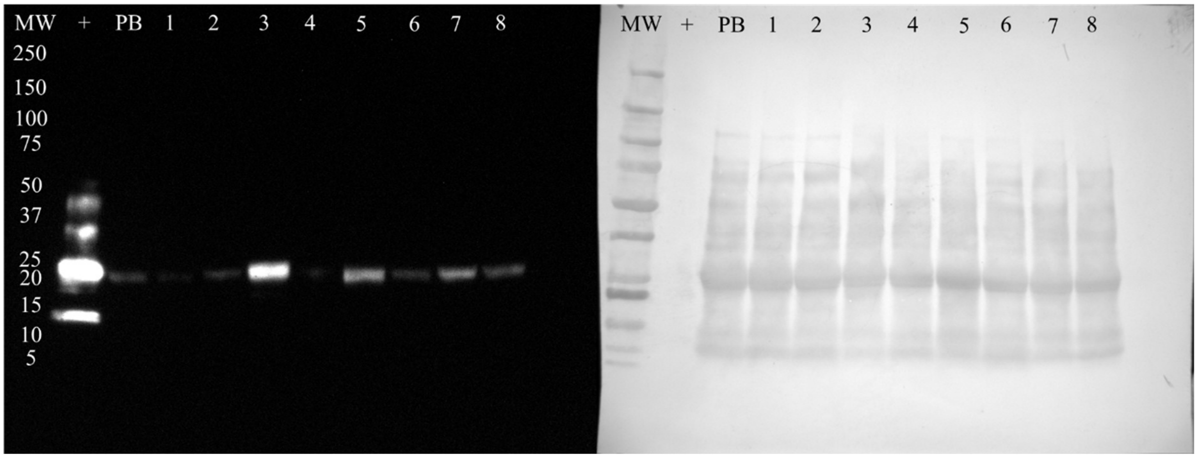

AM.2a

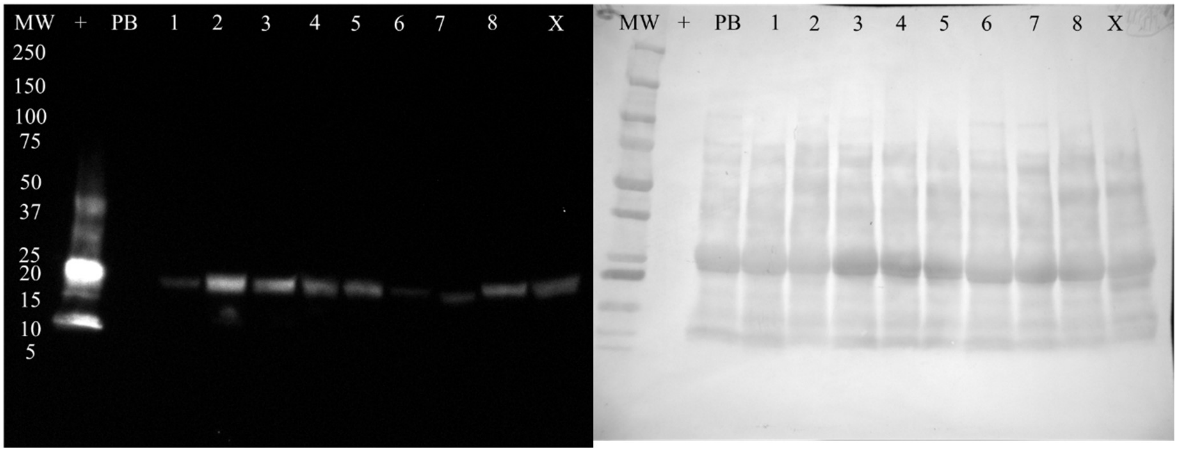
MW + PB 1 2 3 4 5 6 7 8 X
250
150
100
75
50
37
25
20
15
10
5
MW + PB 1 2 3 4 5 6 7 8 X

NR.3

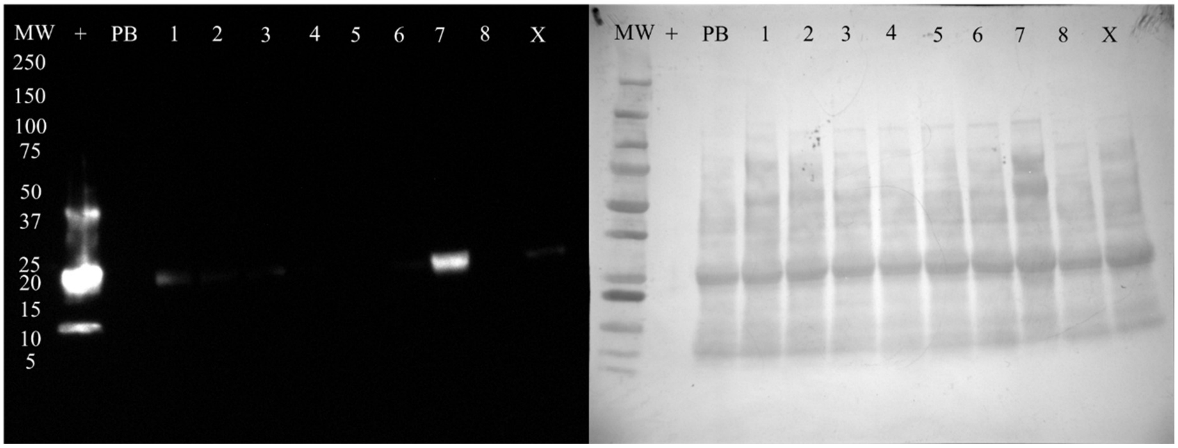
MW + PB 1 2 3 4 5 6 7 8 X
250
150
100
75
50
37
25
20
15
10
5
MW + PB 1 2 3 4 5 6 7 8 X

HO.3

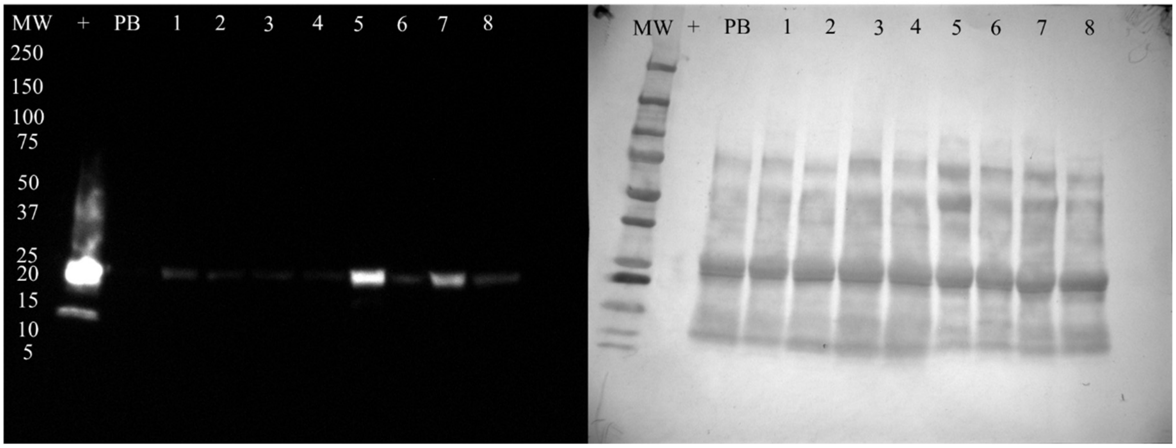
MW + PB 1 2 3 4 5 6 7 8
250
150
100
75
50
37
25
20
15
10
5
MW + PB 1 2 3 4 5 6 7 8

KB.3

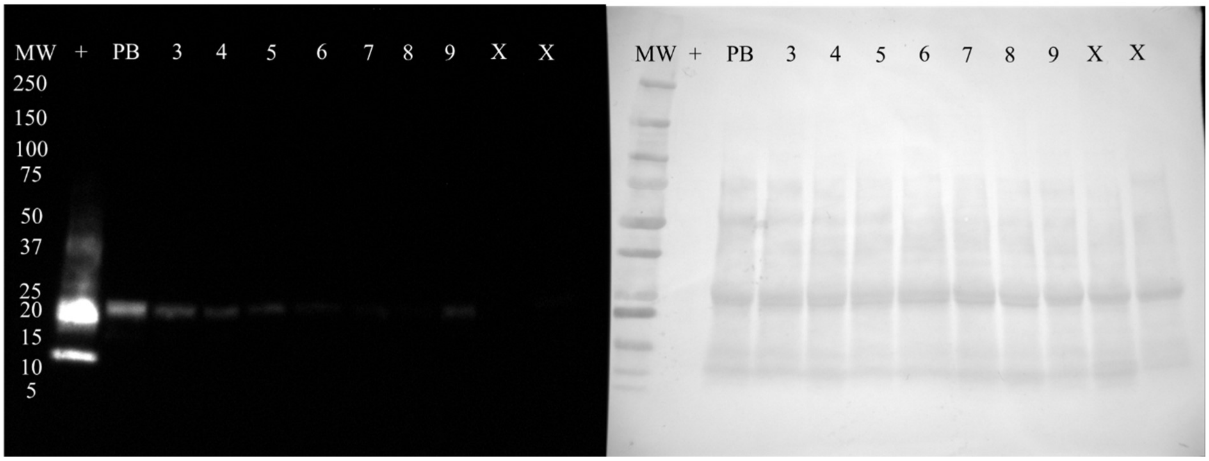
MW + PB 3 4 5 6 7 8 9 X X
250
150
100
75
50
37
25
20
15
10
5
MW + PB 3 4 5 6 7 8 9 X X

SR.3

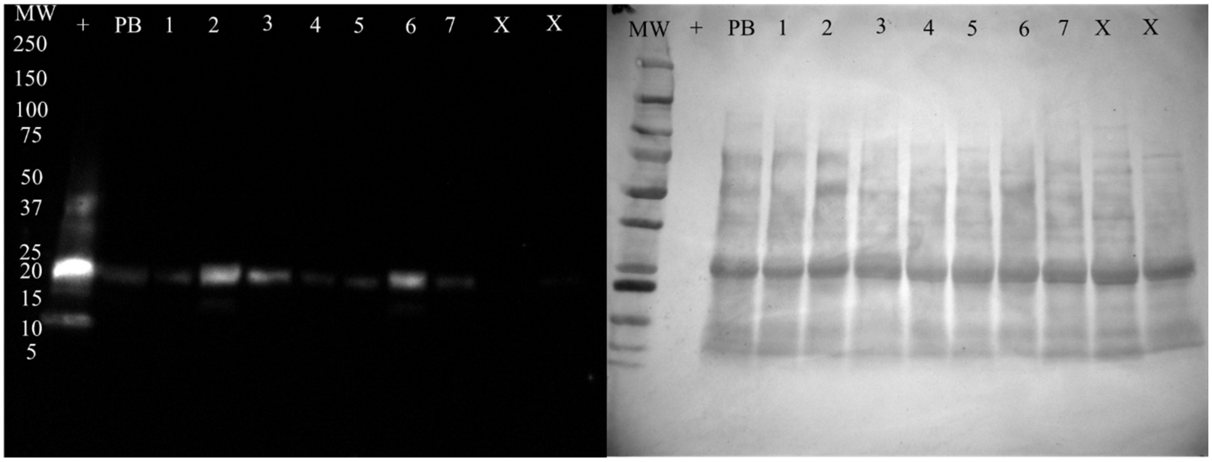
MW + PB 1 2 3 4 5 6 7 X X
250
150
100
75
50
37
25
20
15
10
5
MW + PB 1 2 3 4 5 6 7 X X

OS.3

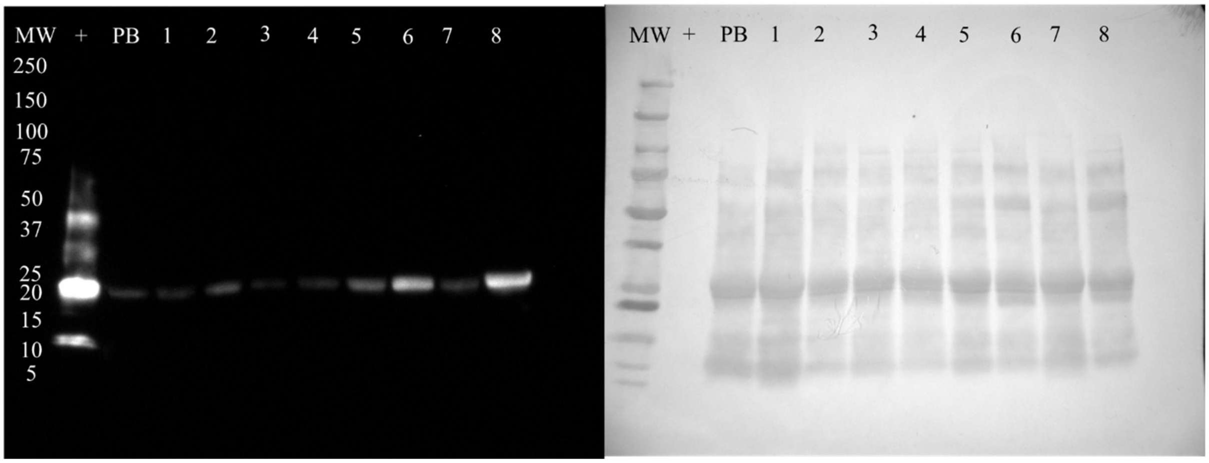
MW + PB 1 2 3 4 5 6 7 8
250
150
100
75
50
37
25
20
15
10
5
MW + PB 1 2 3 4 5 6 7 8

ZZa.1

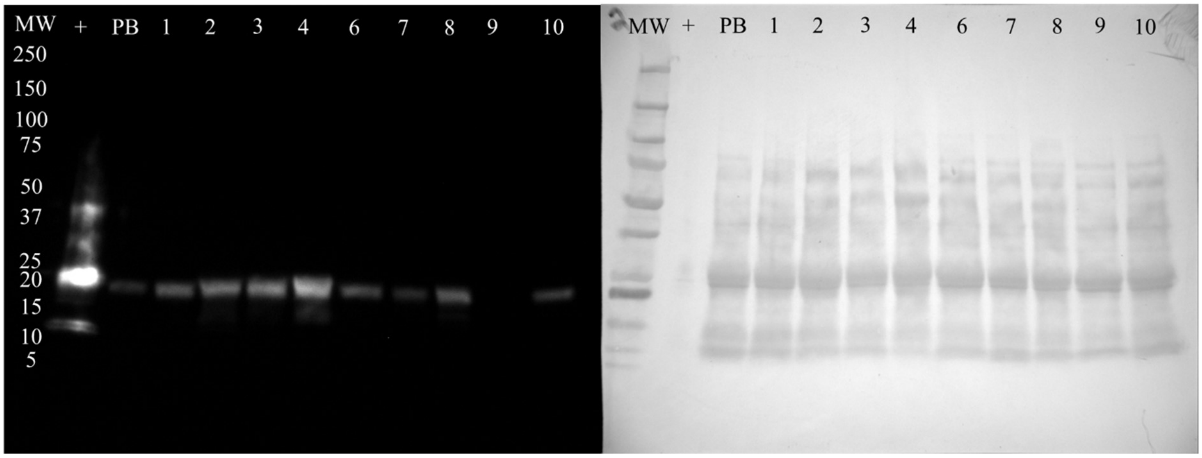

ZZ.2b

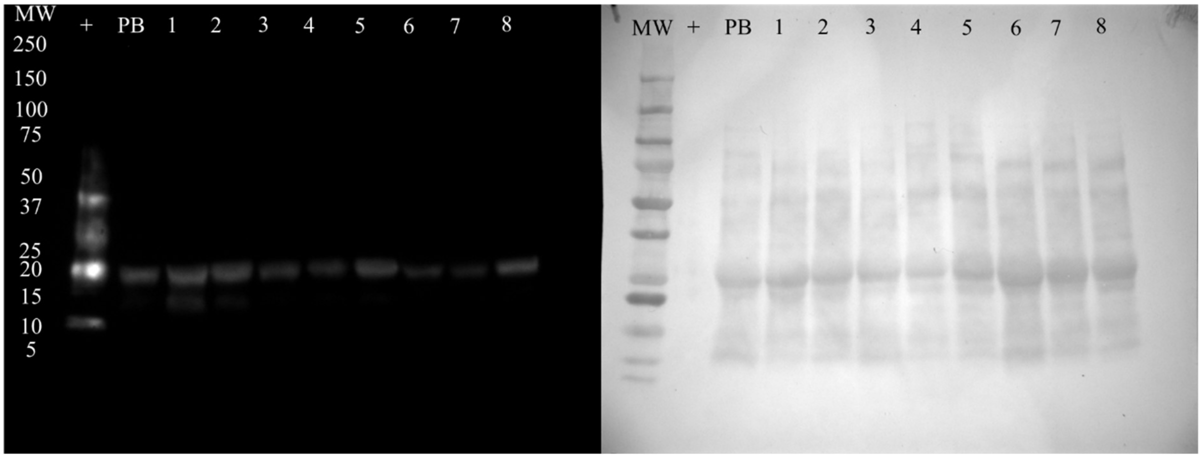

LT.3

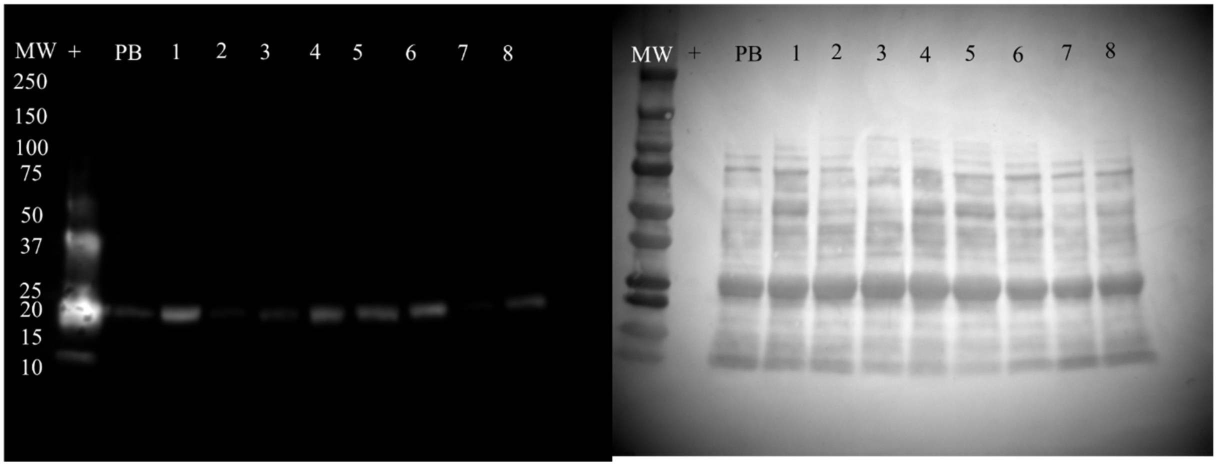

HO.1

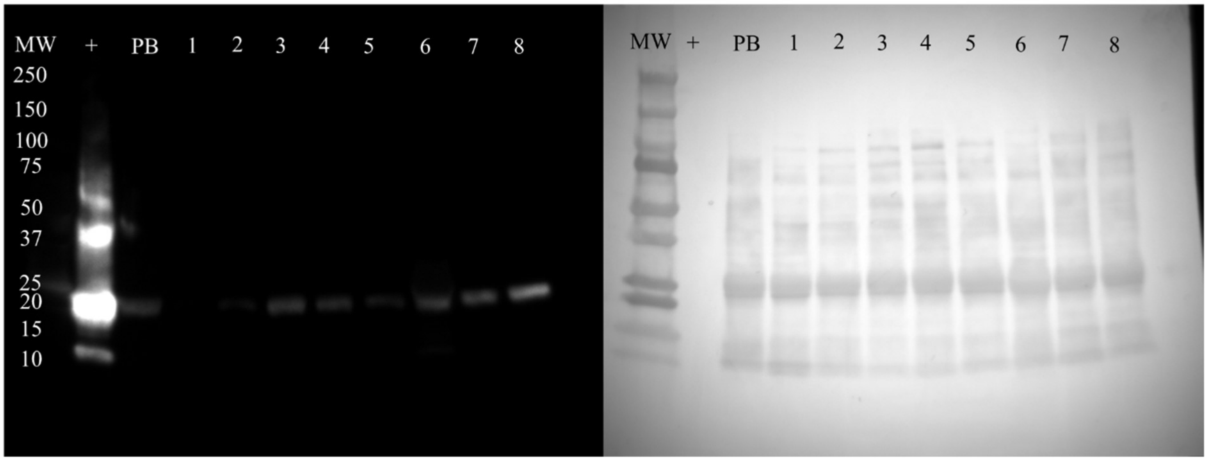

MTb.1

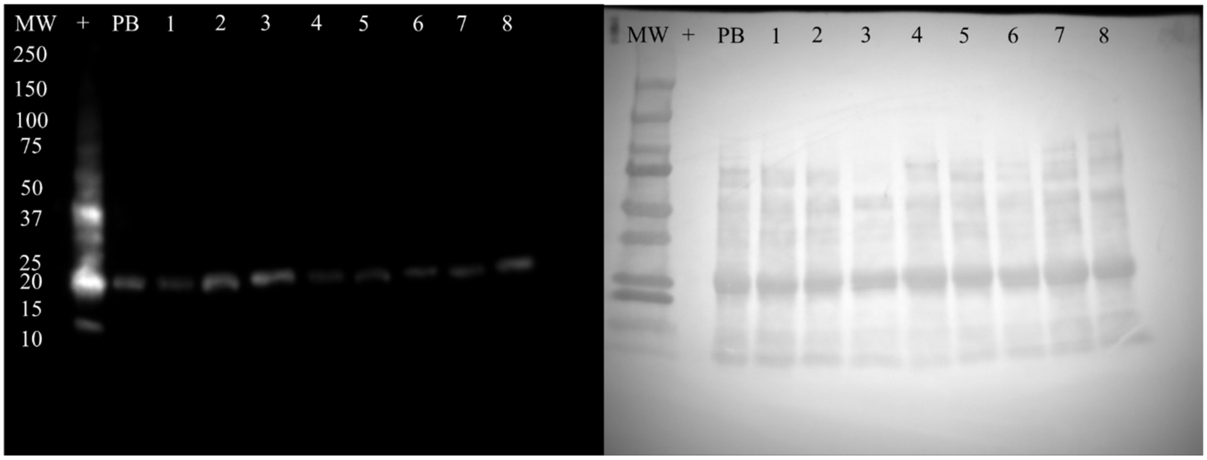

AMa.3

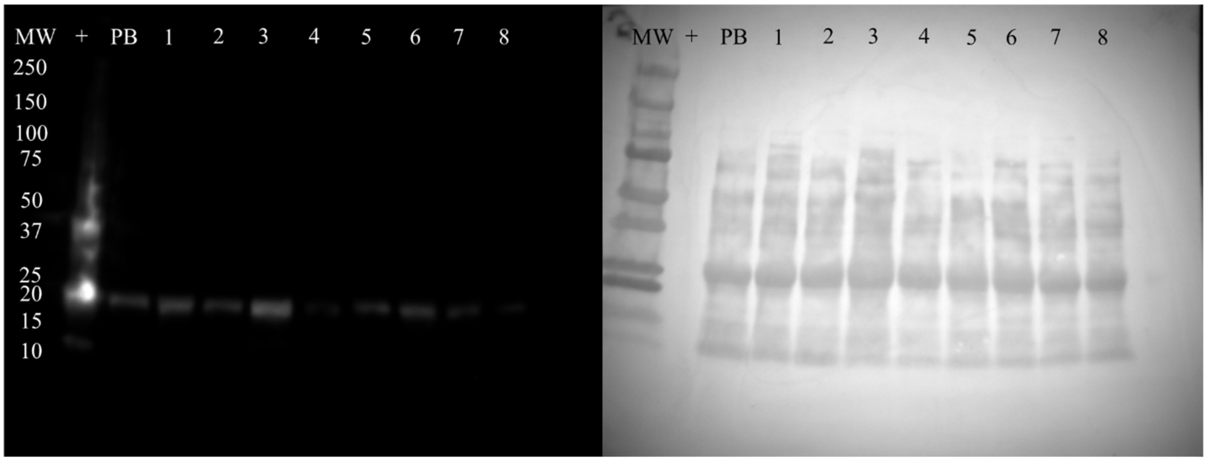

AMb.3

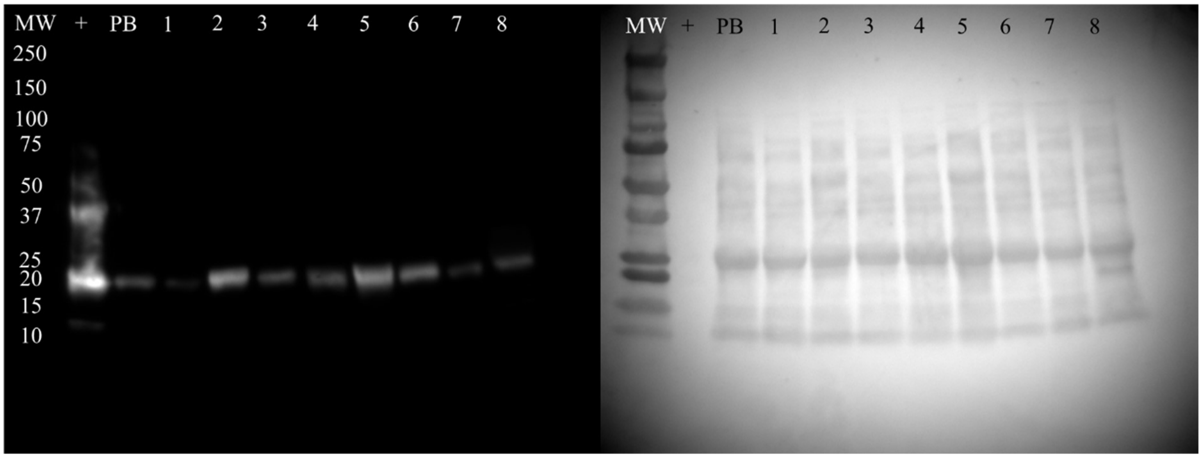

AMc.3

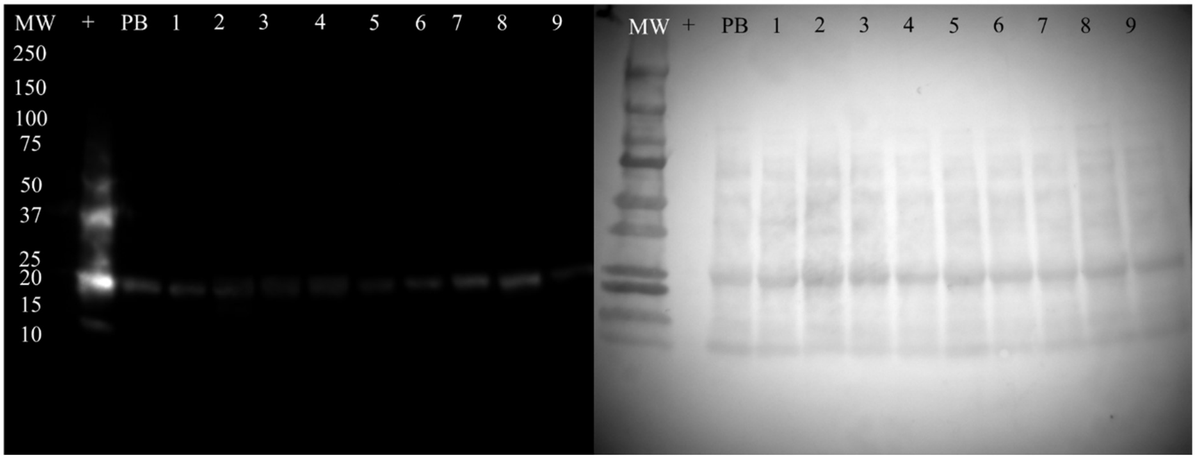

AMd.3

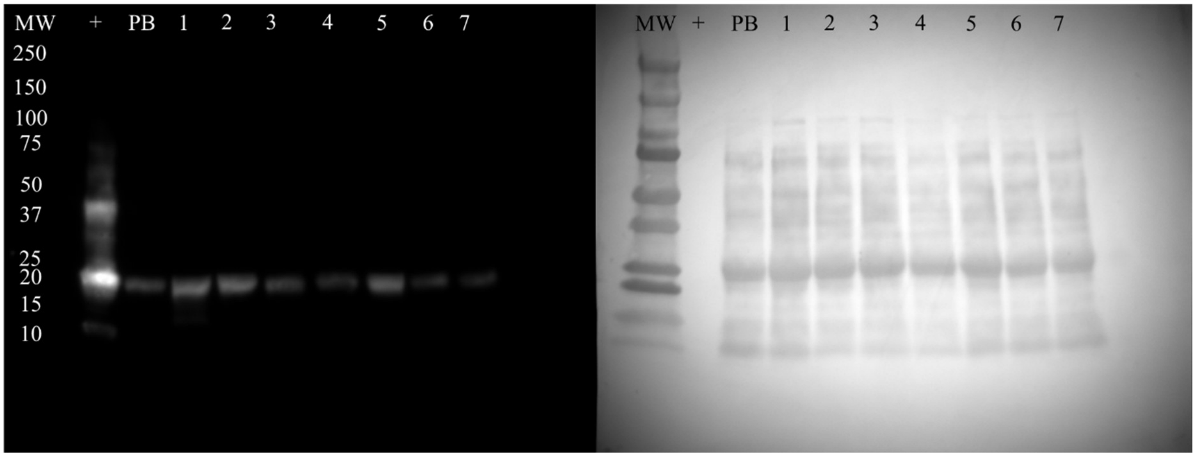

AMe.3

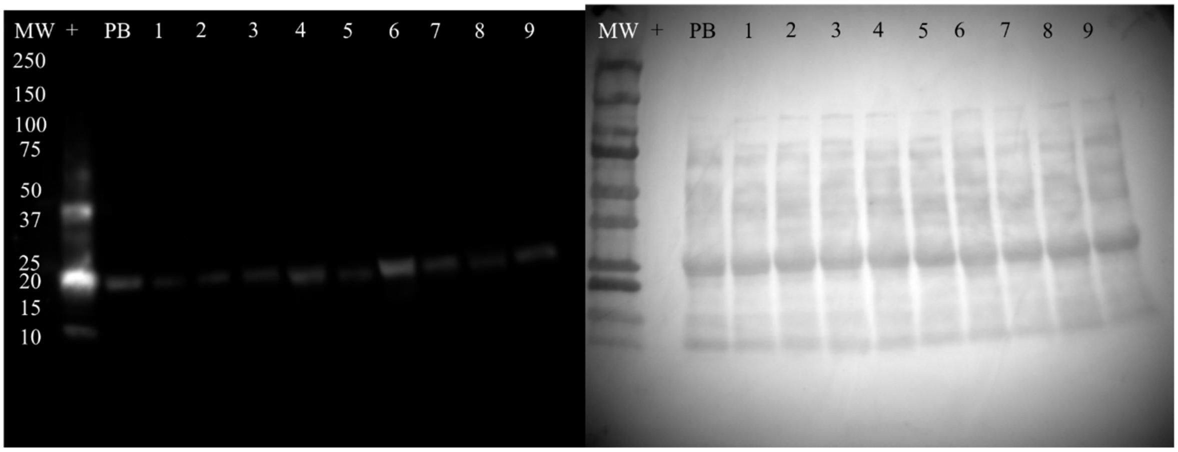

Supplement: S1 File — (PDF) [file pone.0225354.s002.pdf]
